# Rescue endoscopic ultrasound-guided hepaticogastrostomy with antegrade stenting for food-impacted complete papillary occlusion via intentional stent-mesh traversal

**DOI:** 10.1055/a-2777-4777

**Published:** 2026-01-22

**Authors:** Hidenobu Hara, Hikari Ishii, Risa Katsumata, Tomohisa Ashikawa, Kazuomi Sakaki, Kouhei Yoshino, Shinya Sakita

**Affiliations:** 1 53327 Department of Gastroenterology, Yokohama City Minato Red Cross Hospital, Yokohama, Japan

Endoscopic ultrasound (EUS)-guided hepaticogastrostomy with antegrade stenting (EUS-HGAS) provides durable drainage when transpapillary access is not feasible 1 2 , but complete papillary occlusion can prevent guidewire passage. We report a rescue case in which a double-guidewire technique 3 4 enabled intentional traversal of the stent mesh to complete EUS-HGAS.

**Fig. 1:**
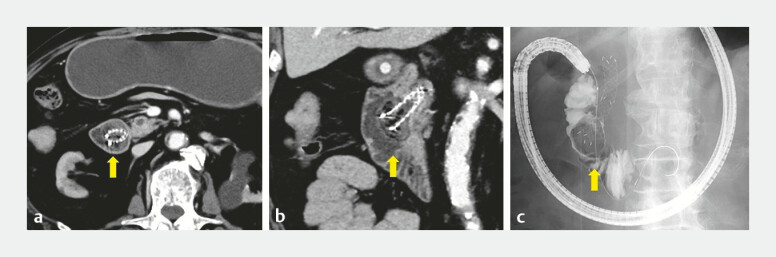
Preprocedural imaging demonstrating a food-impacted papilla with complete obstruction. **a** Axial CT shows dense intraluminal food impacted at the periampullary segment (arrow). **b** Coronal CT confirms the food residue abutting the papilla (arrow). **c** Fluoroscopic duodenography demonstrates a filling defect with contrast hold-up at the papilla (arrow). CT, computed tomography.

A 91-year-old man with pancreatic cancer previously underwent endoscopic retrograde cholangiopancreatography, with the placement of a 10-mm self-expanding metal stent (SEMS) of multi-hole design; a duodenal SEMS was later placed for malignant obstruction. Computed tomography (CT) and contrast studies showed food residue around the papilla, forming an obstructive mass ( Fig. 1 ). The patient developed acute cholangitis, and rescue EUS-HGAS was planned ( Video 1 ).

Bypassing a food-impacted papilla via intentional stent-mesh traversal.Video 1

**Fig. 2:**
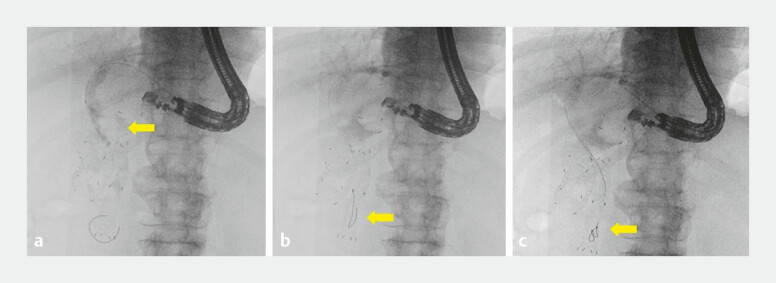
Cholangiography and guidewire attempt before mesh traversal. **a** Cholangiography demonstrates a hilar obstruction. **b** Guidewire passage across the papilla is impeded by food impaction (arrow). **c** A double-guidewire technique was used to attempt the traversal of the impaction, but crossing remained unsuccessful (arrow).

**Fig. 3:**
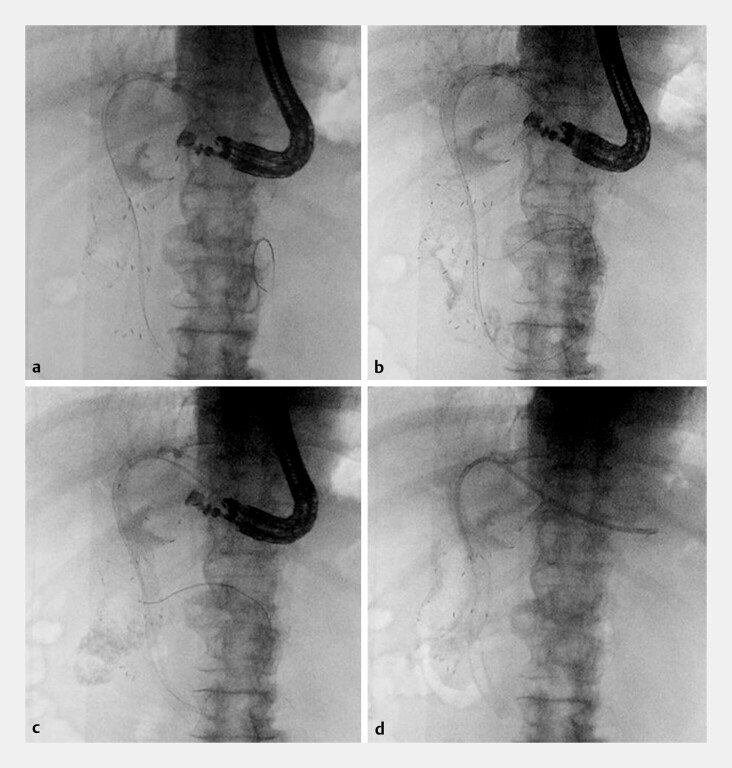
Intentional stent-mesh traversal and completion of rescue EUS-guided hepaticogastrostomy with antegrade stenting (EUS-HGAS). **a** The guidewire intentionally traverses the mesh of the pre-existing transpapillary self-expandable metallic stent (SEMS) and is positioned on the duodenal side. **b** The catheter crosses the SEMS mesh; contrast injection confirms intraduodenal positioning. **c** Two uncovered SEMSs are deployed in series from the duodenum toward the hilar segment to restore luminal continuity. **d** A plastic stent is placed across the HGS fistula to secure the hepaticogastrostomy fistula.

The left intrahepatic duct (B3) was punctured with a 19-gauge FNA needle, and a 0.025-inch guidewire was advanced into the intrahepatic duct. Cholangiography confirmed the intraductal position, and a tapered catheter was inserted. Contrast demonstrated hilar obstruction ( Fig. 2 **a** ). Repeated attempts to cross the papilla with a guidewire failed ( Fig. 2 **b** ), and catheter advancement was impeded by the indwelling stent. A double-lumen cannula enabled a double-guidewire technique with advancement toward the peripapillary segment; yet, crossing remained impossible ( Fig. 2 **c** ). We elected intentional traversal through the stent mesh. The leading-lumen wire crossed the mesh of SEMS ( Fig. 3 **a** ); after failed catheter tracking, reintroduction of the tapered catheter permitted successful mesh traversal. Contrast confirmed the intraduodenal position ( Fig. 3 **b** ). Two uncovered SEMSs (8 × 80 mm and 8 × 60 mm) were deployed in series toward the papillary side to restore luminal continuity ( Fig. 3 **c** ). The HGS fistula was not dilated to minimize the bile-leak risk, and a plastic stent (7 F, 15 cm) was placed from the hepatic duct into the HGS tract ( Fig. 3 **d** ). No complications occurred on clinical/CT follow-up.

To our knowledge, this is the first report of rescue EUS-HGAS overcoming complete papillary occlusion from food impaction around a pre-existing SEMS by stent-mesh traversal.

Endoscopy_UCTN_Code_TTT_1AS_2AD
